# Practitioners' Bookshelf

**Published:** 1893-02-25

**Authors:** 


### THE PRACTITIONERS' BOOKSHELF.

### "THE YEAR BOOK OF TREATMENT."*

The general practitioner requires, in these days of numerous medical publications, some small handy *résumé* of the new discoveries in pathology and treatment made during the year, and he may turn with advantage to Cassell's "Year Book of Treatment." The present volume, the ninth that has been issued, has been improved by the introduction of two new articles, one on "Anæsthetics," compiled by Dr. Dudley Buxton, whose name alone is sufficient to guarantee the excellence of the letter-press; the other, by Professor W. H. Corfield, on "Public Health and Hygiene," "a branch of medicine which is daily increasing in importance and scientific accuracy," as the editor justly remarks in the preface.

The general practitioner in England has little time or opportunity to follow the publications of foreign journals, and we are glad to see the prominence which has been given in the volume before us to the writings of German, French, and American physicians and surgeons.

It is impossible in a short review such as this to enter into details concerning the various articles contained in the book, but we believe the reader will find full information, with references for fuller should he need it, on all branches of surgery and medicine, both as regards treatment and pathology.

The success that has hitherto followed the publication of the "Year Book" will, we feel sure, be continued, for it is just the hand-book that the general practitioner requires, being well printed in clear type, arranged carefully into paragraphs, with headings in thick letters, and is completed with a good index of authors and subjects.

Without wishing to be too critical, we might suggest that the treatment of myxœdema hardly comes under the heading of general surgery, and that the selected list of new books, &c., would be more valuable if the prices were stated.

* "The Year Book of Treatment" for 1893. (London: Cassell and Co.)

